# Beta-human chorionic gonadotropin, carbohydrate antigen 19-9, cancer antigen 125, and carcinoembryonic antigen as prognostic and predictive biological markers in bladder cancer

**DOI:** 10.3389/fonc.2024.1479988

**Published:** 2024-12-23

**Authors:** Hyeong Dong Yuk, Jang Hee Han, Seung-Hwan Jeong, Chang Wook Jeong, Cheol Kwak, Ja Hyeon Ku

**Affiliations:** ^1^ Department of Urology, Seoul National University Hospital, Seoul, Republic of Korea; ^2^ Department of Urology, Seoul National University College of Medicine, Seoul, Republic of Korea

**Keywords:** bladder neoplasma, tumor biomarkers, prognosis, beta-human chorionic gonadotropin, carcinoembryonic antigen

## Abstract

**Introduction:**

We evaluated the prognostic potential of the Beta-human chorionic gonadotropin (β-hCG), Carbohydrate Antigen 19-9 (CA19-9), Cancer Antigen 125 (CA125), and Carcinoembryonic Antigen (CEA) tumor markers for bladder cancer.

**Methods:**

We analyzed the records of 369 patients who underwent radical cystectomy for urothelial cancer (UC) between October 2012 until December 2019. Levels of CA19-9, CA125, CEA, and β-hCG before radical cystectomy were measured in all patient samples, and serum biomarker cutoff values were used as normal and elevated values.

**Results and discussion:**

The proportion of abnormal β-hCG (P<0.001), CA19-9 (P<0.001), and CA125 (P=0.033) was significantly higher in locally advanced bladder UC than in organ-confined bladder UC. In patients with preoperative β-hCG and CA125 abnormality, there was poor prognosis of recurrence-free survival (RFS)(P=0.003, P=0.042) and overall survival (OS) (P=0.003, P=0.002). Using the Cox multivariate regression analysis, both β-hCG (HR: 3.88, 95% CI: 1.43–10.25) and CA125 (HR: 6.21, 95% CI: 1.34–32.16) were found to be significant independent factors for predicting OS and RFS. In addition, patients with a high number of increased tumor markers showed significantly worse OS ((P<0.001) and RFS (P=0.002) than patients with a low number of increased tumor markers. In conclusion, serum β-hCG and CA125 levels could potentially be used for UC prognosis in patients undergoing radical cystectomy. To assess their usefulness in evaluating long-term recurrence and survival, further treatment responses and large-scale additional studies are needed.

## Introduction

Bladder cancer is the eighth most common cancer in men in the United States and the fourth highest cause of cancer death among cancers (1). At initial diagnosis, approximately 75% of patients are diagnosed with non-muscle invasive bladder cancer (NMIBC) confined to the mucosa or submucosa, while 25% of patients are diagnosed with muscle invasive bladder cancer (MIBC) (2).

For patients with NMIBC, a transurethral resection of bladder tumor (TURB) surgery is the standard treatment (3). However, after TURB surgery, approximately 70% of treated patients relapse several times and 10–15% progress to MIBC (3); thus, bladder cancer patients undergo repeated surgeries and tests due to frequent relapses. In addition, patient follow-up is required for long periods of time, making bladder cancer treatment one of the most costly treatments from diagnosis to death, among malignant tumors (3, 4). However, despite the diversity, repetition, and high cost of these treatments, MIBC has a poor prognosis after metastasis occurs. The overall 5-year relative survival rate for bladder cancer is 77% on average. However, if there is local and distant metastasis, the survival rate decreases to 38% and 5%, respectively (1).

Grades or stages of tumors based on pathological biopsies have long served as a major prognostic factor for bladder cancer. However, previous studies have reported that, in the case of preoperative staging with TURB, inaccurate results may be obtained in preoperative overstaging at 28–55% and preoperative understaging at 28–40% (3, 4). In addition, tests that are commonly used to diagnose and track bladder cancer have some disadvantages; urine cytology and CT or MRI scans have limited accuracy and sensitivity, while cystoscopy is invasive.

Monoclonal antibodies are used to detect serum antigens associated with certain malignant tumors (5). For example, it is known that sugar proteins detected in blood by monoclonal antibodies are useful as tumor markers for diagnosing and detecting disease or monitoring treatment response (5). One of the most commonly used serum tumor markers are cancer antigens (5). As mediators of biological activity, cancer antigens are known to be involved in the malignant transformation and progression of tumorous epithelial cells (5). Some cancer antigens include the carbohydrate antigen 19-9 (CA 19-9), carbohydrate antigen 125 (CA 125), and carcinoembryonic antigen (CEA) (5). They are used as serum tumor markers of many malignant tumors. CA19-9 is most frequently used in response to treatment for patients with pancreatic cancer (6). In addition, CA125 and CEA are most commonly used for the early detection of ovarian cancer and colon cancer recurrence, respectively (7, 8). In addition, the β-subunit of human chorionic gonadotropin (β-hCG) is used to diagnose and monitor treatment response to gestational trophoblastic disease and testicular cancer (9, 10).

Considering the high rate of recurrence of bladder cancer and the various types of tests and long-term surveillance for its treatment, it is necessary to consider the availability of measurable markers in serum that can quantitatively change in response to disease progression, relapse, or treatment. Therefore, in this study, we explored the potential of the four aforementioned tumor markers as prognostic factors for bladder cancer.

## Materials and methods

### Study population

We retrospectively analyzed the records of 369 patients who had radical cystectomy with bladder UC from October 2012 until December 2019. Patients with a history of colorectal, pancreatic, and ovarian cancers and related diseases were excluded from this study, in addition to patients who had undergone neoadjuvant chemotherapy. The criteria for study inclusion included patients diagnosed with UC on histopathologic examination of MIBC. Serum samples were taken prior to surgery and were followed up post-surgery.

### Study design

Preoperative levels of CA19-9, CA125, CEA, and β-hCG were measured in all patients. Serum biomarker cutoff values were established based on their respective normal reference ranges (CA19-9: 37 U/mL; CA125: 35 U/mL; CEA: 5ng/mL; β-hCG: 5 mIU/mL). We collected basic patient information, such as age, height, weight, sex, and performance status, and collected histological information such as Tumor-Node-Metastasis (TNM) stage, carcinoma *in situ* (CIS), lymphovascular invasion (LVI), and margin positive status. We also collected a variety of oncologic outcome information, including recurrence, adjuvant and palliative therapy, and mortality. In the absence of recurrence and metastasis, radical cystectomy follow-up was performed every 3 months for 3 years, then every 6 months until 5 years, and (lastly) every year after 5 years. Blood and urine tests, and urine cytology, were performed at each follow-up, and radiographic tests such as Computed Tomography (CT) and bone scans, were performed every 3–6 months for 5 years, and then every year after 5 years.

### Statistical analysis

To determine the statistical significance for each of the four biomarkers (β-hCG, CA19-9, CA125, and CEA), we calculated the required sample size based on a power analysis. Assuming a medium effect size (Cohen’s d = 0.5), a significance level (α) of 0.05, and a power of 0.8, the required sample size was estimated to be approximately 64 participants per group, resulting in a total sample size of 256 participants.

For clinicopathologic information, the study population analyzed the continuous function and the nominal functions using Student’s t-test and chi-square tests, respectively. Continuous variables were expressed as mean values with standard deviation or median values with quartile ranges. Categorical frequency was expressed as a percentage. The primary and secondary evaluation variables of the study were overall survival (OS) and recurrence-free survival (RFS). Kaplan-Meier analysis was used to estimate survival curves, and log-rank tests were used to compare the differences between survival rates. Cox proportional risk regression analysis was used to identify independent predictors of RFS and OS. All statistical tests were performed using IBM SPSS Statistics, Version 24.0 (IBM, Armonk, NY, USA). *P*<0.05 was considered statistically significant.

## Results

A total of 369 patients were included. Their median age was 69 years (Interquartile Range [IQR] 22–88), and 77.2% were male. A total of 53 patients (14.4%) had more than one abnormal tumor biomarker value before radical cystectomy. The number of patients with abnormal tumor biomarkers before radical cystectomy were as follows: 7 (1.9%) with abnormal CEA, 18 (4.9%) with abnormal β-hCG, 19 (5.1%) with abnormal CA125, and 29 (7.9%) with abnormal CA19-9 (**Table 1**).

**Table 1:** Clinicopathological characteristics of the study patients.

Variables	N=369
Mean age (year)	67.3 ± 10.8
Male	285 (77.2%)
Mean BMI (kg/m2)	23.6 ± 3.7
ASA	
1-2	338 (91.6%)
≥3	31 (8.4%)
Mean Charlson comorbidity index	0.7 ± 0.9
Clinical T stage	
≤T2	335 (90.8%)
≥T3	34 (9.2%)
Clincal N stage	
N0	320 (86.7%)
N+	49 (13.3%)
Clincal M stage	3 (0.8%)
M0	366 (99.2%)
M1	3 (0.8%)
Operation type	
ORC	289 (78.3%)
RARC	80 (21.7%)
Diversion type	
Neobladder	256 (69.4%)
Conduit	107 (29.0%)
Continent cutaneous	6 (1.6%)
Pathologic T stage	
≤T2	239 (64.7%)
≥T3	130 (35.3%)
Pathologic N stage	
N0	307 (83.2%)
N+	62 (16.8%)
Lymphovascular invasion	77 (20.9%)
Adjuvant chemotherapy	58 (15.7%)
G-C	51 (13.9%)
DDMVAC	3 (0.8%)
Others	4 (1.0%)
hCG	1.9 ± 12.6
hCG ≤5	351 (95.1%)
hCG >5	18 (4.9%)
CA 125	15.2 ± 28.6
CA125 ≤35	350 (94.9%)
CA125 >35	19 (5.1%)
CEA	6.9 ± 59.1
CEA ≤20	362 (98.1%)
CEA>20	7 (1.9%)
CA19-9	46.1 ± 395.4
CA19-9 ≤ 37	340 (92.1%)
CA19-9>37	29 (7.9%)

The patient group were divided into organ-confined bladder cancer and locally advanced bladder cancer, and were compared to two groups. The ratio of abnormal β-hCG, CA19-9, and CA125 ranged from 3 (1.1%) to 15 (14.3%) for β-hCG, 12 (4.5%) to 17 (16.2%) for CA19-9, and 9 (3.4%) to 10 (9.5%) for CA125; each was significantly different (P <0.001, P<0.001, P=0.033, respectively; **Table 2**).

**Table 2:** Preoperative serum levels of hCG, CA 19-9, CA 125, and CEA according to organ- confined and locally advanced bladder cancer.

	Organ confined	Locally advanced	p- value
	(N=264)	(N=105)	
hCG	0.4 ± 1.4	5.8 ± 23.2	0.019
hCG ≤5	261 (98.9%)	90 (85.7%)	< 0.001
hCG >5	3 (1.1%)	15 (14.3%)	
CA 125	12.2 ± 12.9	22.6 ± 49.0	0.035
CA125 ≤35	255 (96.6%)	95 (90.5%)	0.033
CA125 >35	9 (3.4%)	10 (9.5%)	
CEA	7.3 ± 68.9	5.7 ± 18.6	0.720
CEA ≤20	259 (98.1%)	103 (98.1%)	1.000
CEA>20	5 (1.9%)	2 (1.9%)	
CA19-9	10.7 ± 39.7	135.0 ± 733.4	0.086
CA19-9 ≤ 37	252 (95.5%)	88 (83.8%)	<0.001
CA19-9>37	12 (4.5%)	17 (16.2%)	

**Figure 1:**
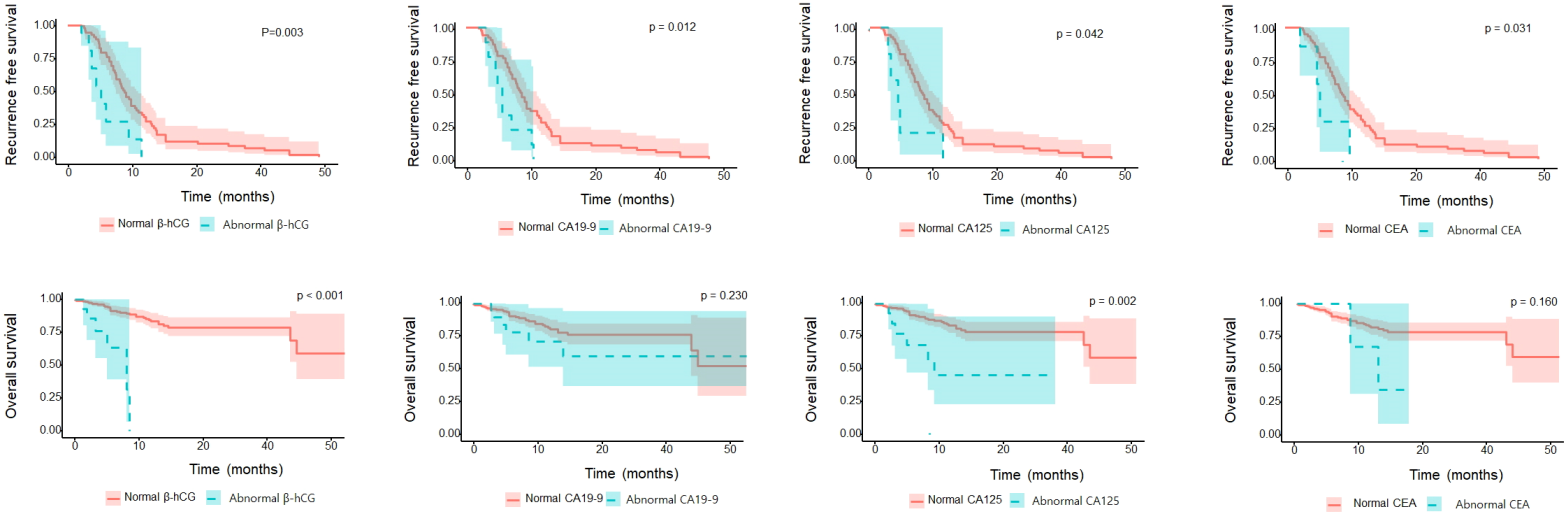
Oncologic survival outcomes according to the β-hCG, CA19-9, CA125, CEA markers.

In assessing patient OS according to each tumor marker, the abnormal β-hCG and CA125 groups had significantly worse OS than the normal β-hCG and CA125 groups (P=0.003 and P=0.002, respectively). However, the abnormal CA19-9 and CEA groups were not different from the normal CA19-9 and CEA groups (P=0.230 and P=0.157, respectively). In assessing RFS according to each tumor marker, the abnormal β-hCG, CA19-9, CA125, and CEA groups had significantly worse RFS than their respective normal groups (P=0.003, P=0.012, P=0.042, and P=0.031, respectively; **Figure 1**).

A multivariate Cox regression analysis was performed to identify significant predictors of patient OS and RFS. Abnormal β-hCG (hazard ratio [HR]: 3.88; 95% confidence interval [CI]: 1.43–10.25), abnormal CA 125 (HR: 6.21; 95% CI: 1.34–32.16), pT3-4 (HR: 4.23; 95%CI: 2.45–7.43), pN1-3 (HR: 4.56; 95%CI: 2.49–8.31), and adjuvant chemotherapy (HR: 3.19; 95%CI: 1.70–5.89) were confirmed as important predictors for OS. In addition, abnormal β-hCG (HR: 4.51; 95%CI: 1.49–12.41), CA 125 (HR: 4.15; 95%CI: 1.39–11.24), pT3-4 (HR: 3.73; 95%CI: 1.92–7.50), and pN1-3 (HR: 1.99; 95%CI: 0.90–4.13) were identified as significant predictors for RFS (**Table 3**).

**Table 3:** Multivariate Cox proportional hazards analyses of beta-hCG, CA19-9, CA125, CEA on overall survival and recurrence free survival.

Variables	Recurrence free survival		Overall survival	
	HR (95%Cl)	P value	HR (95%Cl)	P value
Age	1.01 (0.98-1.04)	0.569	0.98 (0.96-1.01)	0.152
Male	0.68 (0.34-1.45)	0.099	0.78 (0.43-1.46)	0.420
ASA 3-4	0.85(0.20-2.54)	0.791	0.63 (0.18-1.69)	0.630
hCG >5	4.51 (1.49-12.41)	0.005	3.88 (1.43-10.25)	0.006
CA19-9 >37	2.27 (0.08-5.66)	0.095	2.14 (0.89-4.82)	0.074
CA125 >35	4.15 (1.39-11.24)	0.007	6.21 (1.34-32.16)	0.019
CEA >20	3.31 (0.46-15.95)	0.161	1.63 (0.51-4.43)	0.367
pT3-4	3.73 (1.92-7.50)	<0.001	4.23 (2.45-7.43)	<0.001
pN1-3	1.99 (0.90-4.13)	0.043	4.56 (2.49-8.31)	<0.001
Adjuvant chemotherapy	1.60 (0.68-3.44)	0.248	3.19 (1.70-5.89)	0.550

**Figure 2:**
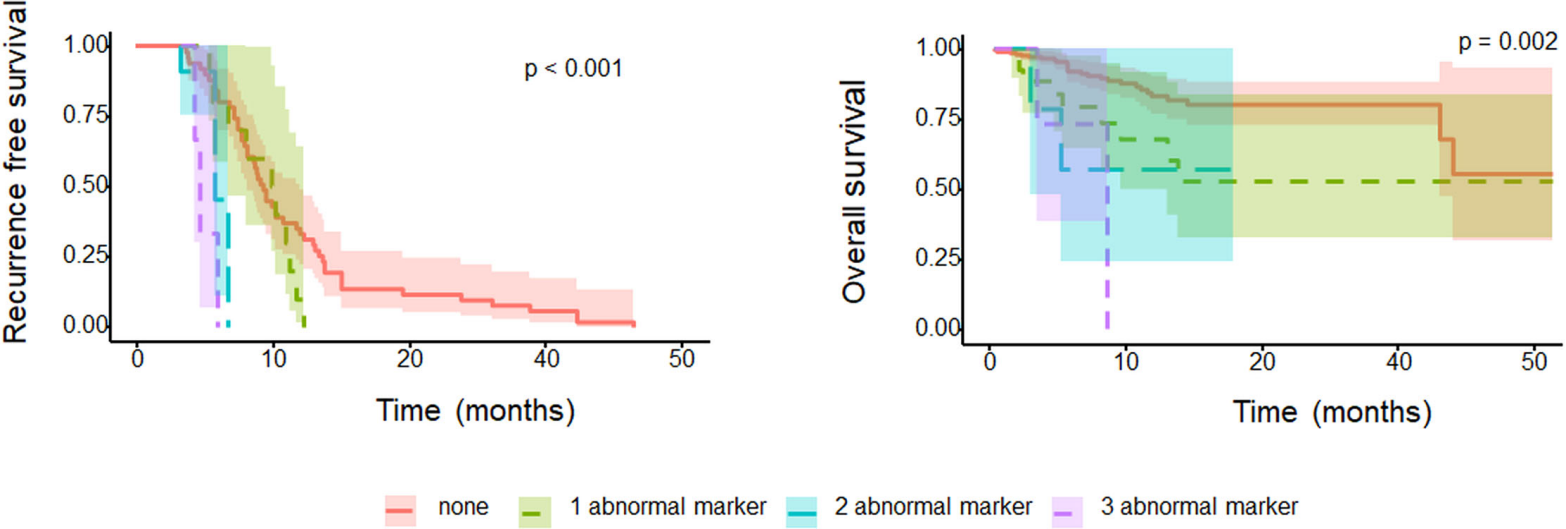
Oncologic survival outcomes according to the number of abnormal tumor markers.

When patients were stratified into three subgroups according to the number of abnormal tumor makers, as the number of abnormal tumor markers used for prediction was higher, there was a significant difference in OS and RFS (P<0.001, P=0.002; **Figure 2**).

Multivariate Cox regression analysis also revealed that the number of abnormal tumor markers was an independent predictor for OS, e.g., one abnormal tumor marker (HR: 1.99; 95%CI: 0.84–4.74), or two abnormal tumor markers (HR: 4.18; 95%CI: 0.94–18.66), or three abnormal tumor markers (HR: 4.59; 95%CI: 0.91–22.66) could independently predict OS. In addition, the number of abnormal tumor markers was also an independent predictor for RFS, e.g., one abnormal tumor marker (HR: 1.23; 95% CI: 0.58–2.63), or two abnormal tumor markers (HR: 5.17; 95% CI: 1.37–19.45) or three abnormal tumor markers (HR: 11.40; 95% CI: 2.77–46.97; **Table 4**) could independently predict RFS.

**Table 4:** Multivariate Cox proportional hazards analyses of the number of abnormal tumor markers on overall survival and recurrence free survival.

Variables	Recurrence free survival		Overall survival	
	HR (95%Cl)	P value	HR (95%Cl)	P value
Age	1.01 (0.97-1.03)	0.627	1.02 (0.98-1.06)	0.383
Male	1.19 (0.63-2.25)	0.594	0.97 (0.45-2.10)	0.940
ASA 3-4	2.05 (0.66-6.27)	0.212	0.32 (0.04-2.78)	0.301
Increased tumor marker		0.002		0.033
1	1.23 (0.58-2.63)	0.590	1.99 (0.84-4.74)	0.115
2	5.17 (1.37-19.45)	0.015	4.18 (0.94-18.66)	0.035
3	11.40 (2.77-46.97)	0.001	4.59 (0.91-22.66)	0.031
pT3-4	2.48 (1.21-5.09)	0.013	5.10 (2.16-12.04)	<0.001
pN1-3	2.36 (1.17-4.77)	0.016	4.38 (2.03-9.46)	<0.001
Adjuvant chemotherapy	1.24 (0.88-1.78)	0.218	1.36 (0.90-2.05)	0.144

## Discussion

In our study, we focused on the potential of the CA19-9, CA125, CEA, and β-hCG tumor markers as prognostic factors for bladder cancer. The proportion of abnormal β-hCG, CA19-9, and CA125 was significantly higher in locally advanced bladder urothelial carcinoma (UC) than in organ-confined bladder UC, and in patients with preoperative β-hCG and CA125 abnormality, OS and RFS were poor. In addition, in the Cox multivariate regression analysis, β-hCG and CA125 were found to be significant independent factors for predicting bladder cancer prognosis. When we divided the patients according to the number of abnormal tumor markers, patients with a high number of multiple abnormal tumor markers showed worse OS and RFS than patients with a lower number of tumor markers. **Figure 2** effectively illustrates the correlation between the number of abnormal tumor markers (0, 1, 2, or 3) and the survival outcomes of bladder cancer patients following radical cystectomy. The OS (Overall Survival) graph indicates that patients with higher numbers of abnormal markers show significantly reduced survival rates, with those having two or more abnormal markers exhibiting the poorest OS. This trend underscores the value of these markers as independent indicators of survival risk.

Similarly, the RFS (Recurrence-Free Survival) graph demonstrates that patients with a greater count of abnormal tumor markers face higher recurrence risks, resulting in decreased RFS. Each additional abnormal marker level corresponds to an increased recurrence risk, emphasizing the utility of these markers in predicting recurrence and guiding more vigilant follow-up strategies for high-risk patients. Together, these graphs highlight the prognostic relevance of β-hCG, CA19-9, and CA125 levels in patient monitoring and tailored post-surgical care.

Cancer antigens and cell adhesion molecules are generally known to be involved in epithelial invasion or immune system evasion of epithelial cells during tumor progression and malignant transformation (11, 12). Cancer antigens, such as CA19-9, CA125, and CEA, are serum cancer biomarkers commonly used in various types of cancer. These markers help predict the prognosis and response to relapse treatment due to changes in serum levels.

CA19-9 is associated with chemotherapy recurrence, treatment response, and prognosis in patients with pancreatic cancer. High CA19-9 is associated with poor survival and chemotherapy response (13, 14). In addition, CA19-9 levels are known to increase in hepatocellular carcinoma, esophageal cancer, colon rectal cancer, and gastrointestinal cancer (15). Kurokawa et al. reported that the concentration of serum CA19-9 increases with the volume of UC tumor tissues (16). Margel et al. reported that increased CA19-9 before radical cystectomy was associated with poor survival and was specifically reported as an independent predictor of CSS (HR1.5, 95%CI 1.1–2.3, *P*= 0.02) (17). In our study the percentage of patients with abnormal CA19-9 in the locally advanced group was 4.5%, whereas that in the organ-confined group was 16.2%, respectively (*P*<0.001), and the preoperative abnormal CA19-9 group related to poor RFS (*P*=0.012). However, CA19-9 was not significant as an independent predictor in our study.

In our study, increased abnormal CA125 and β-hCG were associated with poor prognosis.

The secretion of β-hCG by bladder cancer cells is thought to stimulate tumor growth. *In vitro* studies have shown that β-hCG can promote urothelial carcinoma cell proliferation in a dose-dependent manner. Additionally, when cells are treated with anti-β-hCG antibodies, this stimulatory effect is inhibited, suggesting that β-hCG plays a role in cancer cell growth (18).

CA125 is a glycoprotein that is primarily expressed in the epithelial cells of the reproductive organs, but it is also produced by malignant urothelial cells in the bladder. In bladder cancer, particularly in advanced stages, the shedding of epithelial cells and the production of mucin by these cancer cells can lead to elevated CA125 levels in the serum and urine (19).

Kouba et al. reported that 35% of patients with abnormal CA125 had higher regionally advanced disease (pT4 or N+) than those with organ-confined UC (≤pT2N0) (20). In addition, CA125 was reported to be abnormal in all non-resectable patients (20). Chang et al. reported an increase in CA125 in approximately 11% for preoperative high grade or invasive UC, but reported no significant association between CA125, RFS, and tumor grade (21). However, the increase in CA125 in locally advanced UC was reportedly correlated with OS (*P*<0.001) (21). CA125 expression in various tumors of epithelial origin is known to promote tumor invasion via tumor cell growth and increased cell motility. It is also known to be involved in cell-to-cell interaction, which enables metastasis (22) while chemotherapy is known to reduce the sensitivity of cancer cells (23). Limited research has been performed on the relationship between β-hCG and UC. Venyo et al. reported that immunohistological expression of β-hCG in UC is related to NMIBC recurrence and poor OS in MIBC (24). Douglas et al. argued that β-hCG levels were significant in determining the therapeutic effect of chemotherapy in UC patients (25). Β-hCG is known to play an important role in the stages of carcinogenesis, such as transformation, angiogenesis, and immune evasion (26). Sun et al. reported that an increase in β-hCG causes an increase in tumor cell population because of a decrease in tumor cell death (27).

The clinical significance of this study is that beta-hCG and CA125 can play a crucial role in the early identification of high-risk bladder cancer patients. While these biomarkers may not be relevant for all bladder cancer patients, elevated levels of β-hCG and CA125 in certain individuals can indicate potential metastatic disease or early progression of cancer that may not be detected through other diagnostic tests. This can enable more proactive treatment or closer monitoring for such patients.

Despite the interesting findings, our study has some limitations. First, being a retrospective study, there may be selection bias. However, tumor marker serum tests were conducted with prior study planning, and since 2016, we have established a prospective database for radical cystectomy patients, which likely reduced bias. Second, the cohort size was small, particularly for subgroups with abnormal biomarker levels like β-hCG (18 patients) and CA125 (19 patients). This limited sample size reduces statistical power and raises concerns about the reliability of the results. Previous studies have reported abnormal tumor markers in about 10% of patients, while our study found abnormal levels in only 5–7%. A larger sample size would be necessary to validate our findings. Finally, our results are based on data from a single institution, limiting generalizability. Multicenter studies will be crucial to confirm the clinical relevance of our findings.

## Conclusions

Elevated serum levels of β-hCG and CA125 were found to be associated with poorer overall survival (OS) and recurrence-free survival (RFS) in patients with urothelial carcinoma undergoing radical cystectomy. These markers may provide valuable prognostic information, helping to identify high-risk patients for more tailored monitoring and treatment. To assess their usefulness as a tumor marker for evaluating long-term recurrence and survival, further treatment responses and large-scale additional studies are needed.

## Data Availability

The raw data supporting the conclusions of this article will be made available by the authors, without undue reservation.
